# Dominance in a socially dynamic setting: hierarchical structure and conflict dynamics in ravens' foraging groups

**DOI:** 10.1098/rstb.2020.0446

**Published:** 2022-02-28

**Authors:** Palmyre H. Boucherie, Mario Gallego-Abenza, Jorg J. M. Massen, Thomas Bugnyar

**Affiliations:** ^1^ Department of Behavioural and Cognitive Biology, University of Vienna, Vienna, Austria; ^2^ Konrad Lorenz Forschungsstelle, Core Facility for Behaviour and Cognition, University of Vienna, Vienna, Austria; ^3^ Animal Behaviour and Cognition, Department of Biology, Utrecht University, Utrecht, The Netherlands

**Keywords:** dominance structure, fission–fusion dynamic, elo-rating, cognition

## Abstract

Dominance hierarchies typically emerge in systems where group members regularly encounter and compete for resources. In birds, the ‘open’ and dynamic structure of foraging groups may prevent the emergence of structured hierarchies, although this assumption have hardly been tested. We report on agonistic data for ravens *Corvus corax*, collected over two 18-month periods for 183 marked individuals of a wild (fluid) population and 51 birds from six captive (stable) groups. We show that the dominance structure (steep and transitive) in wild foraging groups is strikingly similar to that found in captivity. In the wild, we found that higher ranks are mainly occupied by males, older and more aggressive individuals that also tend to receive fewer aggressions. Exploring the mechanisms sustaining the wild dominance structure, we confirmed that males are more aggressive than females and, with age, tend to receive fewer aggressions than females. Males that are about to leave the foraging groups for some months are less aggressive than newcomers or locals, while newcomers are specifically targeted by aggressions in their first year (as juveniles). Taken together, our results indicate that the socially dynamic conditions ravens face during foraging do not hinder, but provide opportunities for, using (advanced) social cognition.

This article is part of the theme issue ‘The centennial of the pecking order: current state and future prospects for the study of dominance hierarchies’.

## 1. Introduction

Competition for resources and reproduction is a key challenge for animals, and in particular gregarious species [1]. The establishment of dominance hierarchies can (partly) alleviate the costs of competition by regulating and mitigating conflicts [2]. Many social species form dominance relationships as a direct outcome of repeated agonistic interactions, depicting asymmetries in opponents' winning abilities [2]. Functionally, dominance relationships regulate the priority of access to resources [3] and social interactions [4], and can affect individuals’ physiology and fitness [5]. The organization, or structure, of dominance relationships defines the hierarchy [6], according to which individuals can be ranked from the most dominant(s) to the most subordinate(s), as described in the seminal paper on pecking order by Schjelderup-Ebbe [7].

While dominance relationship is a relative and dyadic measure (not a property of individuals), dominance rank refers to an individual's position in the hierarchy [2]. Across species, animals rely on a range of mechanisms to establish and maintain dominance relationships and the associated rank structure [8], varying in complexity. Individuals can, for instance, base their decisions on whether to aggress or submit to a conspecific on the physical appearance of the opponent (e.g. sex, age, body condition [9]) and/or spatial association patterns (close associations are typical for affiliates that could act as allies in conflicts). Coupled with good learning and memory skills, these decision rules could evolve into rule-of-thumb strategies like ‘aggress those that are physically inferior to you’ or ‘aggress those that have not been seen in spatial association lately’. These cognitively simple strategies could become particularly efficient with additional rules like ‘keep on aggressing former victims' or ‘redirect aggression to bystanders’ (i.e. serial and redirected aggressions). Such behavioural patterns might be used selectively according to context, resulting in a flexible adjustment to social situations [10].

In some species, we can also expect animals to individually recognize conspecifics and memorize their own dominance/submission status relative to them. In the latter case, individuals may additionally come to mentally represent the dominance order and infer their own and others' positions, based on transitivity [11]. Both cognitive building blocks, individual recognition and transitive inference, have been successfully demonstrated in experimental studies across taxonomic orders, e.g. paper wasps ([12]; see also [13]), primates, birds and fish [14,15]. In such systems, rank-related aggression strategies may thus emerge, such as individuals directing aggression towards opponents of similar competitive ability, likely to maintain their rank, resulting in a close-competitor strategy [16,17].

Finally, in societies structured by different types of affiliated relationships, ranks may become dependent on the assistance/presence of individuals like kin, partners or friends [18]. Such species are hypothesized to develop a third-party understanding, i.e. they represent not only their own relationships but also the relationships between others [19]. There is unequivocal experimental evidence for third-party understanding in non-human primates [20,21], and various observations have indicated a strategical use of this knowledge, i.e. planning alliances or preventing others from gaining rank [22]. Similar observations exist in some other taxonomic groups (e.g. hyenas [23], horses [24], corvids [25], geese [26]), but experimental tests for third-party understanding in species other than primates are rare and results are mixed [27–30]. In species expressing high degrees of fission–fusion dynamics, it may become difficult for individuals to keep track of their own and others’ relationships [31]. Having the opportunity to leave and join other (sub-)groups might also promote conflict avoidance and dispersive strategies over those of conflict resolution [31]. In comparison with when group composition is stable, highly dynamic social conditions might thus render the emergence and maintenance of structured hierarchies less likely [31,32]. Empirical studies on how dominance hierarchies work under high fission–fusion dynamics are scarce, however (but see [32,33]; see also [34], on the dynamics of dominance).

Common ravens, *Corvus corax*, are an interesting model species to study dominance under ‘complex’ dynamic social conditions: on one hand their foraging groups are characterized by moderate to high fission–fusion dynamics, on the other hand they are structured by age, breeding status and differentiated relationships. Foraging ravens tend to aggregate on ephemeral but rich and monopolizable food sources (e.g. carcasses, anthropogenic food sources like garbage dumps or game parks [35]), forming ‘open’ groups with individuals joining and leaving within and across days [36]. While ravens show high levels of mobility and flexibility in exploiting food sources, they may also develop preferences for particular foraging sites, resulting in almost daily visits to those sites [37]. Despite high degrees of fission–fusion dynamics, subsets of individuals may thus more regularly meet than others at certain locations [38,39]. At our study site, most birds in a foraging group are non-breeders i.e. sexually immature (±70%), or adult but lacking a partner and/or territory (±25%), while territorial breeders are in the minority (±5%) [40]. Ravens are long-term socially monogamous: pair partners stay together over several years, often for life; they form a close affiliative relationship and jointly defend a territory for breeding [35]. Interestingly, pair-bond-like relationships can also be found in non-breeders, typically among males and females (sometimes future mated partners) but also among same-sex partners, often kin (e.g. siblings) or familiar individuals [41–43]. These relationships resemble pair-bonds in the nature and frequency of their association and affiliation patterns [41–43]. Pair partners like non-breeder affiliates often act as allies in conflicts, typically when foraging [37,44,45].

We analysed 12 datasets of agonistic interactions collected within a monitoring programme on wild and captive ravens in the course of 12 years. In a first step (objective 1), we examined the structure and certainty of the dominance rank hierarchies under dynamic social conditions in the wild and compared them with the relatively stable social conditions in captivity. Specifically, we used two datasets of 18 months on a total of 183 individually marked ravens belonging to a wild population in the northern Austrian Alps, and 10 datasets from our captive colony of 51 ravens housed in six social groups ranging from 6 to 11 individuals. We tested the assumption that the constraints posed by fission–fusion dynamics (difficulties in track-keeping of relationships, opportunity for dispersive conflict avoidance) should result in a dominance structure different from that found in captivity. Previously, Braun & Bugnyar [37] argued that physical appearance (sex and age) and/or spatial associations (as typical for bonded birds) may serve as reliable cues for ravens to broadly categorize individuals into being ‘dominant’ or ‘subordinate’ under dynamic free-flight conditions. They more specifically proposed that individuals could follow the rules-of-thumb that: males dominate females (owing to their weight, around 1250 g for males versus 1100 g for females; see also [41]), older birds dominate younger birds (owing to their weight and/or experience) and bonded birds dominate non-bonded birds (owing to social support). They further argued that birds of similar physical appearance and/or bonding status might develop dominance rank hierarchies within their social category. We thus tested the hypothesis that structured hierarchies do not form in raven foraging groups at the whole group level but may exist within categories of similar individuals (e.g. of a certain sex or age), resulting in a step-wise pattern in the hierarchy. The findings from the wild should differ from those in captivity, where we expected to find structured (steep and transitive) hierarchies at the whole group level [28,44,46] owing to stable social conditions and limited conflict avoidance options. After establishing the hierarchy structure in the wild, we investigated patterns sustaining ranks, considering in particular conflict dynamics (i.e. how much the individuals initiate and receive aggressions) along with individuals' age and sex. In line with the theory [37,41], we expected older males and more aggressive individuals to dominate the hierarchy.

In a second step (objective 2), we examined how these conflict dynamics were affected by the open and dynamic nature of ravens’ foraging groups, notably by the high variation in how often and how long individuals are present/absent at the foraging site. Firstly, we expected ravens with long presence (locals) to initiate more and receive fewer aggressions than non-local birds (e.g. ‘newcomers’ or individuals that have left the local group for months), as the local dominance structure should be particularly salient for ravens that frequently visit the site. Returning birds could actively try to reintegrate into the dominance structure (and eventually regain their previous rank) and could be specifically targeted by local birds with similar social status. This pattern might be most pronounced in adult males, as we expected them to dominate females and younger birds in the hierarchy. Males might also be more aggressive than females since they are physically stronger, whereas females might be more often victims of aggressions. We used our two wild datasets to test these predictions.

## 2. Methods

### (a) General methods

#### (i) Field conditions and sampling methods of wild ravens

In the course of our long-term monitoring programme (established in 2007) of a wild raven population in the northern Austrian Alps, we caught more than 400 birds (mean: 27 per year) with drop-in traps [47]. Caught birds were measured, blood-sampled for sex and kinship analysis, and marked with a combination of coloured rings and wing tags for individual identification (electronic supplementary material, S1). Age was determined via the colour of the tongue and oral cavity, which changes from pink to black with maturation [48] (electronic supplementary material, S1). As the ravens' socio-cognitive development is strongest in the first 2 years [46], we considered the following age classes: juvenile (1–12 months), subadult year 2 (13–24 months), subadult year 3 (25–36 months) and adult (more than 36 months). Adults range between 1 and 14 years old in this foraging group. From 61% of the marked juveniles we have records exceeding the first summer, on average for 4.1 years per bird. From 76% of the subadults and 90% of the adults, we have records over consecutive years, on average for 4.1 and 5.1 years.

We studied ravens in the area of the Cumberland Wildpark (latitude: 47.807° N, longitude: 13.950° E), an Alpine Zoo with hiking paths and enclosures of native animals situated in the river valley of Grünau im Almtal. Ravens use the park for foraging in the enclosures when the park's animals are fed [49]. Their foraging groups are composed primarily of non-breeders and typically range from 20 to 80 birds, whereof about 50% can be identified individually. Since 2007, we have recorded almost daily the identity and social interactions (agonistic and affiliatory; collected ad libitum during 30 min observation sessions) of the marked ravens present during the morning feedings of wild boars (*Sus scrofa*), brown bears (*Ursus arctos*) and wolves (*Canis lupus*). Age structure and sex ratio within foraging groups have been fairly constant over years (around 30% juvenile, 40% subadult, 30% adult; the male : female ratio per age class varies between years, but stays around 40 : 60%). Yet, we can see a large variation in how often and how regularly individuals join the feedings (ranging from a few days per year to more than 300 days per year; [37]).

#### (ii) Wild study periods

We analysed two 18-month datasets compiling agonistic and affiliative data on two distinct wild foraging groups. The first dataset (Wild1) includes 89 marked individuals, sampled between September 2008 and February 2010 by one observer; and the second (Wild2) includes 100 individuals sampled between September 2017 and February 2019 by a team of field assistants. Of the 189 individuals present in the two datasets, 3% were present in both; we thus worked with a total of 183 independent individuals. See electronic supplementary material, S2 for further details on dataset characteristics, sampling methods and sample sizes.

#### (iii) Housing conditions, sampling methods and study periods of captive ravens

We analysed 10 captive datasets, collected from six groups (ranging from 6 to 11 individuals), all housed in large outdoor aviaries (160–240 m^2^) at the Haidlhof Research Station (Bad Vöslau, Austria) and at the Cumberland Wildpark. Groups were all composed of non-breeders (i.e. sexually immature birds in their first years) but differed in respect of the birds' origin and upbringing (parent- or hand-raised). While some captive individuals were involved in affiliative relationships (typically with one to three birds), some had no affiliative interactions, which compares well with the situation found in the wild [50]. Across groups, data were collected using either 30 min ad libitum sampling in food monopolization experiments (three datasets), 5 min focal sampling (five datasets), or 30 min ad libitum sampling in a neutral context (two datasets). In the two latter cases, data were collected from January to June for four datasets and from July to December for the three others (electronic supplementary material, table S2).

### (b) Methods, objective 1: dominance hierarchies

#### (i) Datasets and conflict definition

Analyses were run separately on the two wild and 10 captive datasets. For the wild datasets, we selected individuals that were seen in more than 10% of all observation sessions (Wild1: 52 marked ravens, 275 sessions; Wild2: 50 marked ravens, 386 sessions). Analyses of sampling effort and data sparseness of all datasets, wild and captive, indicated sufficient sampling to ensure a reliable estimation of the hierarchy (see electronic supplementary material, S3). We used directed–decided conflicts, defined by an initial aggression (for which the identities of the aggressor and the victim are known), and a clear outcome i.e. the victim leaves/retreats from, or submits to, the aggressor (detailed ethogram in electronic supplementary material, S4).

#### (ii) Dominance hierarchy structure

We used the randomized elo-rating method developed by Sánchez-Tójar and colleagues to infer the hierarchy and evaluate its steepness and uncertainty (R package aniDom v. 0.1.5; [51,52]; see also [53]). Like other elo-rating methods, the randomized elo-rating works on winner–loser sequences, but replicates the initial sequence *n* times, randomizing the order of conflicts (replications were set to 1000). Mean individual ranks and 95% confidence intervals are then inferred from the 1000 individual elo-scores. We evaluated the hierarchy steepness from the visualization of the ‘shape’ of the hierarchy, plotting the probability for a dominant to win a conflict, according to the rank difference with its opponent. In very steep hierarchies, this probability quickly increases to 1, while in flat or unpredictable hierarchies, it would remain close to 0.5 (random) [51]. We quantified the uncertainty of the inferred hierarchy by two means: the repeatability of the individual elo-ratings across randomizations (function ‘estimate_uncertainty_by_repeatability’) and the correlation score between the two inferred hierarchies when splitting the dataset into two halves (function ‘estimate_uncertainty_by_splitting’). Repeatability scores above 0.65 and 0.9 suggest intermediate to very high levels of steepness and a low uncertainty of the inferred hierarchy, respectively. The same logic applies for correlation scores above 0.5 and 0.9, respectively [51].

We evaluated the triangle transitivity as a measure of the orderliness of the dominance structure using the package ‘compete’ v. 0.1 (function ‘ttri_test’, [54]), following the algorithm and code described by Shizuka & McDonald [55]. In transitive hierarchies, if A dominates B and B dominates C, then A dominates C. The function returns a scaled index of triangle transitivity (*t*_tri_) which evaluates the tendency of triadic relationships to be ordered, i.e. transitive [55]. This metric ranges from 0 when the proportion of transitive triangle in a network is not different from random (proportion evaluated as 0.75), and 1 when all triangles are transitive [55]. The associated *p*-value evaluates whether the tested empirical dataset is more ordered (i.e. proportion of transitive triads) than expected by chance.

#### (iii) Daily affiliation ratio and vagrant–resident index

The daily ratio of affiliation was computed to approximate individual bonding status (higher ratio indicating paired individuals and/or individuals with one or several affiliated partners). We did so by dividing the total frequency of affiliations an individual initiated and received by the number of feeding events at which it was present, for each 18-month study period. Affiliations included: contact–sit, allopreening, body contact, allofeeding, co-feeding, co-manipulations, object transfer and play (detailed ethogram in electronic supplementary material, S4). We also computed a vagrant–resident index, as the ratio between the total number of feeding events at which a bird was present and the total number of feeding events at which for each study period. This index ranged from 0 for highly vagrant birds to 1 for highly resident birds.

#### (iv) Rank predictors

We finally investigated how rank (evaluated over an 18-month period) was affected by individuals' sex, age range (see detailed categories below), daily affiliation ratio (covariate), vagrant–resident index (covariate), and the daily ratio of initiated (covariate) and received aggressions (covariate) over the study period. Ranks varied from 1 to *N* (number of individual) in each period, and were inferred for each individual from its mean elo-scores across the 1000 randomizations. Age ranges over the 18-month study period, respectively, corresponded to individuals that hatched: during the study period, 1–10 months old (age range 1); the year before, 5–22 months old (1–2); 2 years before, 17–34 months old (2–3); 3 years before, 29–46 months (3–4); or more (adults). We ran a linear mixed model (LMM, function ‘lmer’, lme4 R package v. 1.1.27.1, [56]), adding the dataset identity (Wild1, Wild2) as a random intercept in the model. We applied Satterthwaite's approximation of degrees of freedom to compute the *p*-values (function ‘tab_model’ option ‘p.val’ = ‘satterthwaite’ in R package sjPlot v. 2.8.9.1; [57]).

### (c) Methods, objective 2: conflict dynamics in groups with changing composition

With this second objective, we further examined the conflict dynamics underlying the wild dominance structure (i.e. initiated and received aggressions). Analyses were performed on a monthly basis to include individuals' temporal variations in presence at the foraging site. Therefore, the daily affiliation ratio and vagrant–resident index were this time computed per month.

#### (i) Datasets

We focused on the two 18-month wild datasets (Wild1 and Wild2). We worked on initiated and received aggressions for which the identity of the aggressor and/or victim was known, respectively (detailed ethogram in electronic supplementary material, S4).

#### (ii) Presence dynamics and data subset

On a monthly basis, we evaluated individuals’ presence status, whereby a bird was scored as ‘present’ if it had been seen in at least 10% of the monthly observation sessions (feeding events). We subsequently categorized individuals' presence dynamics, differentiating periods of ‘arriving’ (i.e. first two months of presence, after at least two months of absence), ‘staying local’ (i.e. present after at least two months and for at least two more months) and ‘before leaving’ (i.e. last two months of presence, before at least two months of absence; full details on the procedure in electronic supplementary material, S5). We worked with a total of 53 (Wild1: 275 sessions) and 64 (Wild2: 386 sessions) marked ravens, for which the monthly presence dynamics were known (in total: 82 arriving, 794 local and 115 leaving individuals).

#### (iii) Statistical analyses

We investigated how the monthly frequency of initiated (model 1) or received (model 2) aggressions was affected by individuals' sex, age class (juvenile, subadult year 2, subadult year 3, adult), daily affiliation ratio (covariate) and presence dynamics (arriving, before leaving, staying local). Since we worked with count response variables i.e. behavioural frequencies, the vagrant–resident index (covariate) was simply used this time as a measure of the proportion of time in the study to control for varying observational effort across individuals. We also considered the interactions between: sex and presence dynamic, age and presence dynamic, sex and age, and sex and daily affiliation ratio. We ran two generalized linear mixed models (GLMMs) using a negative binomial distribution and log-link function (function ‘glmer.nb’, lme4 R package v. 1.1.27.1), to account for the over-dispersed distribution of our dependent variables. To account for pseudo-replication and repeated measures across individuals and time periods we added the individual identity and the year and month when the data were collected as random intercepts in the models. See electronic supplementary material, S6 for general information on statistics and data visualization.

## 3. Results

### (a) Objective 1: dominance hierarchies

#### (i) Dominance structure

**Figure 1.**
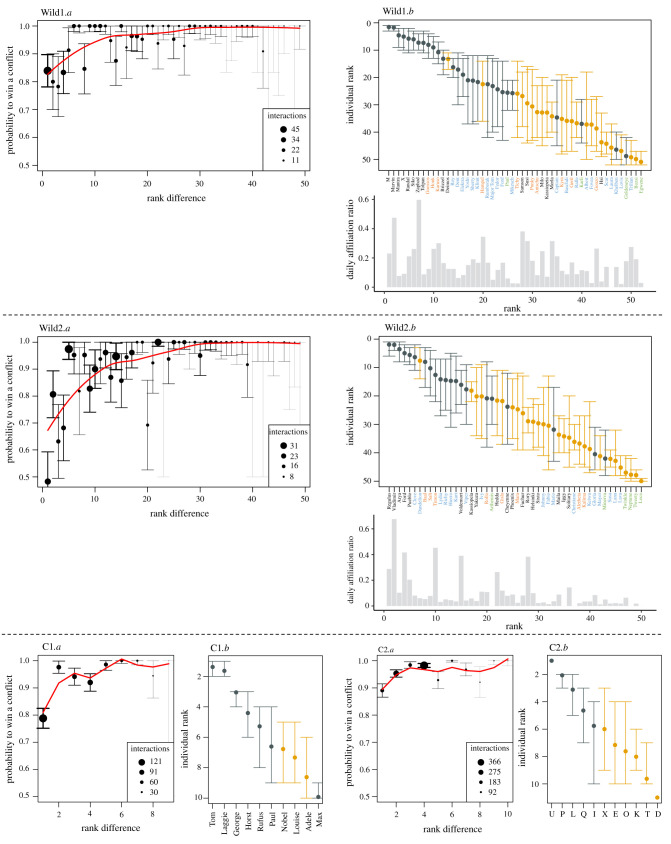
Shape of the hierarchy (*a*) and individuals' dominance rank (*b*) for the two wild (Wild1, Wild2) and two captive datasets (C1 and C2; see all 10 captive datasets in electronic supplementary material, S7). The shape of the hierarchy plots the probability (from 0 to 1) for a dominant to win a conflict with respect to the rank difference with its opponent; point size is function of the number of interactions available in the dataset for each rank difference. Dominance ranks are ordered from top (upper left) to bottom; points represent individuals’ mean rank (inferred from the individual elo-scores) and whiskers the 95% confidence interval across the 1000 randomizations; they are coloured in grey for males and yellow for females. In the two wild populations, individuals' names (on the *x*-axis) are coloured according to their age range over the study period: green for age range 1; blue for 1–2; orange for 2–3. Individuals of the 3–4 age range and adults are coloured in black. Individuals’ daily affiliation ratio (computed over the whole study period) is shown below their respective dominance ranks and is computed as the total sum of affiliations initiated and received for the whole study period, divided by the total number of feeding events when individuals were present. Age range is not depicted for the captive groups, as group members typically hatched in the same year.

We found steep and rather steep dominance structures for both the captive and the wild datasets (see groups summary in electronic supplementary material, table S2). In captivity the probability for a dominant to win a conflict very quickly increased above 0.9 for higher rank differences between the two opponents, and above 0.8 in the wild (figure 1: C1.*a* and C2.*a*, Wild1.*a* and Wild2.*a*; electronic supplementary material, S7 for a complete results overview of all captive datasets). For all datasets, the repeatability scores across randomizations were above 0.8 in captivity (ranging from 0.81 to 1.00 across groups; electronic supplementary material, S3), and equal to 0.93 (Wild1) and 0.91 (Wild2) in the wild. The correlation scores between the two inferred hierarchies (when splitting each dataset into two halves) were above 0.74 in captivity (ranging from 0.74 to 0.97 across groups), and 0.76 (Wild1) and 0.75 (Wild2) in the wild. Together, these scores indicate intermediate to very high steepness and a low uncertainty of the inferred hierarchies in both captive and wild data sets. Finally, the triangle transitivity indices were above 0.91 for all captive datasets expect one (ranging from 0.91 to 1.00 across groups, except C7: 0.60; electronic supplementary material, S3), and equal to 0.96 (Wild1) and 0.82 (Wild2) in the wild, indicating highly transitive hierarchies. Top rank positions were occupied by males in both captive and wild datasets. But in several of the captive groups and in the two wild datasets, some females were also seen in the top half of the hierarchy (i.e. from the most dominant to the average rank; figure 1: C1.*b* and C2.*b*, Wild1.*b* and Wild2.*b*; electronic supplementary material, S7 for a complete results overview of all captive datasets).

#### (ii) Rank predictors

**Figure 2.**
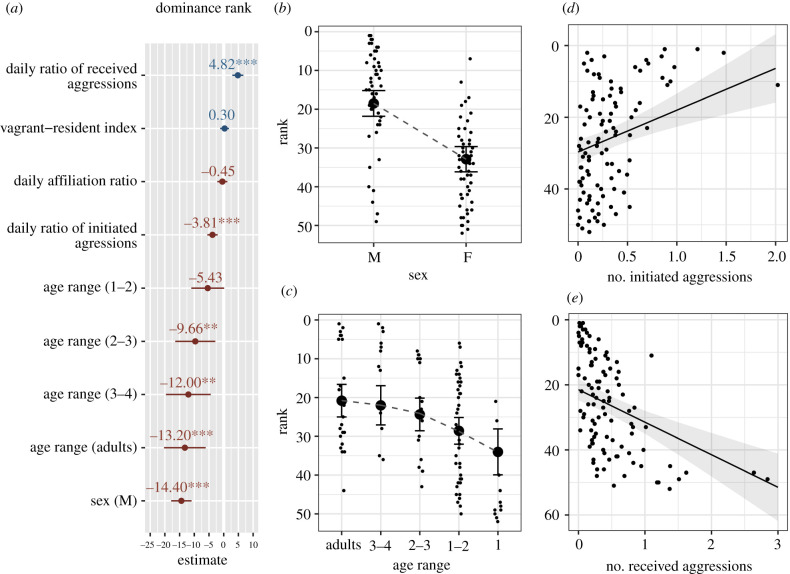
(*a*) Estimates forest plots of the linear mixed model investigating individual rank, together with the associated effects of (*b*) individuals' sex, (*c*) age range, (*d*) daily ratio of initiated aggressions, and (*e*) daily ratio of received aggressions over the study period on individual rank (predicted values), generated with the ‘ggeffect’ function in R package ggeffects [58]. Reference levels for the categorical predictors in the forest plot are respectively: sex (F, female), age range (1). Full model output in electronic supplementary material, S8. Asterisks indicate the level of statistical significance: ***, <0.001; **, <0.01; *, ≤0.05. In (*b*) to (*e*) the *y*-axis is reversed to show top ranks at the top of the graph and match figure 1. Error bars in (*b*,*c*) and shaded area in (*d*,*e*), respectively, represent 95% confidence intervals around the estimated marginal means and the marginal effect regression lines. (Online version in colour.)

Analysing ranks estimated for 102 individuals over two 18-month periods (with two individuals present in both periods), we found that rank was mainly affected by the sex and age of the individual, with males and older individuals, respectively, occupying significantly higher ranks than females and younger birds (estimates forest plot in figure 2*a*, see also figure 2*b,c*, full model output in electronic supplementary material, S8). To a lesser extent, individual aggressiveness and received aggressions were also found significant, with high-rank individuals initiating significantly more aggressions while they tended to be less often the target of aggressions than low-rank individuals (figure 2*a,d,e*, electronic supplementary material, S8).

### (b) Objective 2: conflict dynamics in groups with changing composition

#### (i) Initiated aggressions

**Figure 3.**
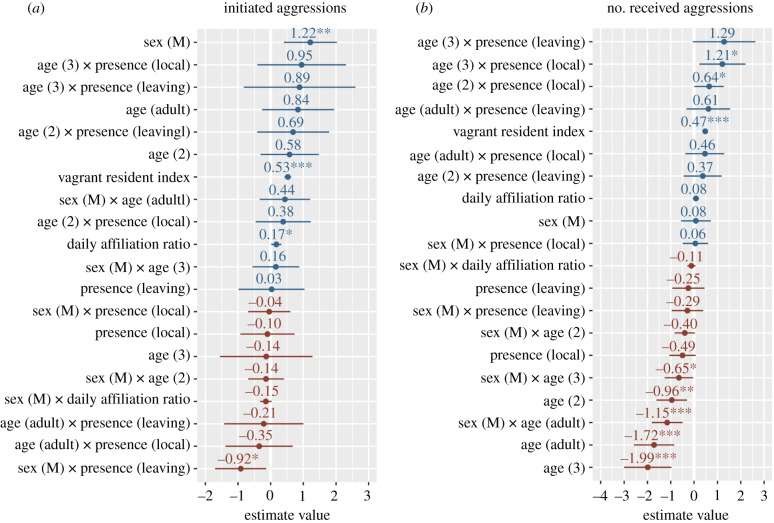
Estimates forest plots of the two generalized linear mixed models investigating monthly rates of (*a*) initiated and (*b*) received aggressions. Reference level for the categorical predictors are respectively: sex (F, female), age (1), presence dynamic (arriving). Asterisks indicate the level of statistical significance: ***, <0.001; **, <0.01; *, ≤0.05. Full model output in electronic supplementary material, S9. (Online version in colour.)

**Figure 4.**
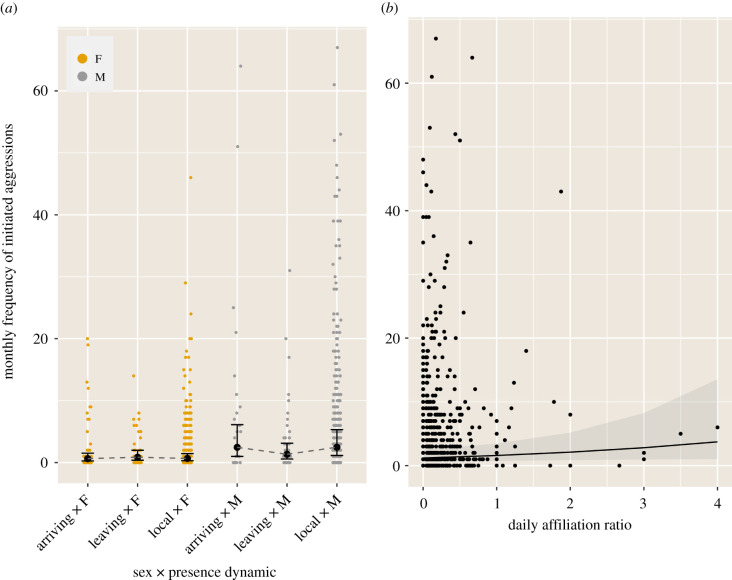
Modelled effects of (*a*) individuals' sex in interaction with the presence dynamic (arriving, leaving, local), and (*b*) daily affiliation ratio on individuals’ monthly frequency of initiated aggressions (predicted count values), generated with the ‘ggeffect’ function in R package ggeffects [58]. Error bars in (*a*) and shaded area in (*b*), respectively, represent 95% confidence intervals around the estimated marginal means and the marginal effect regression line. (Online version in colour.)

Analysing a total of 4048 initiated aggressions over two 18-month periods for 117 marked individuals (with two individuals present in both periods), we found significant effects of sex, the interaction between sex and presence dynamics, and to a lesser extent the daily affiliation ratio (estimates forest plot in figure 3*a*; full model output in electronic supplementary material, S9). While males generally initiated more conflicts than females (figure 4*a*), the difference between sexes was particularly marked for local and ‘arriving’ (newcomers, or birds arriving after having been away from our foraging groups for two months or longer; figure 4*a*). To a lesser extent, individuals' aggressiveness tended to increase with increasing daily affiliation ratio, for all sexes and age classes (figure 4*b*). Note that individuals’ aggressiveness significantly increased with increased vagrant–resident index; however, this is mainly explained by the proportion of time in the study accounted by this predictor (figure 3*a*; electronic supplementary material, S9).

#### (ii) Received aggressions

**Figure 5.**
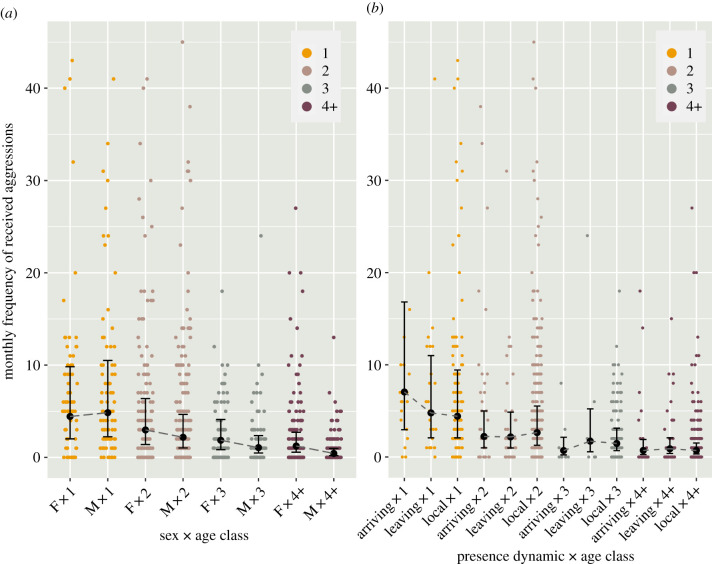
Modelled effects of the interaction between individuals' (*a*) sex and age class (1, 2, 3, 4+ i.e. adult), and (*b*) age and presence dynamic (arriving, leaving, local) on individuals’ monthly frequency of received aggressions (predicted count values), generated with the ‘ggeffect’ function in R package ggeffects [58]. Error bars in (*a*) and shaded area in (*b*), respectively, represent 95% confidence intervals around the estimated marginal means. (Online version in colour.)

Analysing a total of 3847 received aggressions revealed significant effects of age class, the interaction between sex and age class, and to a lesser extent the interaction between age and presence dynamics (estimates forest plot in figure 3*b*; full model output in electronic supplementary material, S9). The amount of aggressions received decreased with age, whereby juveniles (1 year old) received more aggressions than older individuals (figure 5*a*,*b*). Except in juveniles, males tend to receive fewer aggressions than females (figure 5*a*). The effect of presence dynamics differed in age classes: while juveniles received the most aggressions in the first two months after ‘arriving’ (i.e. when integrating with the foraging group for the first time, or after having been away for more than two months), 3-year-old subadults tended to receive slightly fewer aggressions in that period, while the presence dynamics did not affect the amount of aggressions received by adults and 2-year-old subadults (figure 5*b*). Note that the frequency of received aggressions significantly increased with increased vagrant–resident index; however, this is again mainly explained by the proportion of time in the study accounted by this predictor (figure 3*b*; electronic supplementary material, S9).

## 4. Discussion

### (a) Dominance hierarchies

Our findings show that raven groups are structured by a steep and transitive dominance hierarchy, irrespective of the dynamic nature of foraging groups in the wild, and irrespective of the group composition, sampling methods and raising style (parent- or hand-raised) in captivity. Against our hypothesis, the picture obtained from wild ravens falls within the range seen in captivity. For all datasets, captive and wild, the repeatability and correlation scores were well above the theoretical thresholds, indicating low uncertainty—thus a robust assessment—of each inferred hierarchy. We found the same results in the wild in two distinct periods that were 7 years apart and in which only 3% of the identified birds remained the same. This suggests that such a steep and transitive dominance structure is a characteristic feature of wild raven foraging groups, at least under the conditions faced in Middle Europe [59]. Our results are in line with, at that time relatively speculative, interpretations from observations at garbage dumps in Switzerland [40]; how well they fit to ravens in areas with few anthropogenic food sources remains to be tested.

Our robust finding of a structured dominance hierarchy, not only in captivity but also under dynamic conditions in the wild, fits with the competitive nature of socially foraging ravens [35,41], and is in line with primate socio-ecological models [60–63]. Ravens' food competition is mainly characterized by contest competition, which in opposition to scramble competition occurs when a defensible (clumped) food resource can be monopolized by some individuals. Following primate socio-ecological models, species experiencing contest competition are more likely to establish strong linear hierarchies ([60–63]; but see in elephants [64,65] and vampire bats [66]). These models, however, were primarily established to explain the sociality of females, which in most primate species live in stable cohesive groups.

In less cohesive species, fission–fusion dynamics are often interpreted as a strategy to alleviate the costs of foraging competition ([67]; but see also [68,69] for the mitigating effect of predation pressure and travel costs [70] on grouping patterns). Fission–fusion dynamics typically allow dispersive conflict management and reduce scramble competition and/or the intensity of contest competition [31,62,71,72]. Ultimately, this might limit the likelihood for steep and linear dominance structures to develop [31,73,74]. Primates species expressing a high degree of fission–fusion dynamics indeed tend to show low numbers of intra-group aggressions and little evidence or mixed results regarding the emergence of linear and steep hierarchies (spider monkeys [71,75,76], chimpanzees [22,77], hamadryas baboons [78]; but see [72,79,80]). However, linear hierarchies can be found in other fission–fusion societies, typically characterized by a high degree of relationship differentiation (e.g. spotted hyenas [81], elephants [64,65]). In the case of ravens, groups that form at rich and defensible food sources lead to severe contest competition [35,82], which might foster the development of dominance structures. Additionally, if fission allows conflicts to be reduced, fusion events and increased party size on the contrary might increase conflicts, in particular at high-quality food sources and among members of different communities [72]. Future studies should thus aim to compare intra-group (here emerging communities) and inter-group aggressions in ravens, together with the dynamics of fission and fusion events.

### (b) Ranks and conflict dynamics in changing group composition

In line with theory [37,41], our analyses show that rank was mainly affected by sex and age, with males and older individuals occupying higher ranks in the hierarchy. On top of these individual attributes, higher ranks were also associated with higher initiated frequencies and lower received frequencies of aggressions. Corroborating these results, our analyses of conflict dynamics confirm that males were more active than females in initiating conflicts (see also [37]), and thus higher ranked. Also in line with rank predictors, older birds tended to receive fewer aggressions, males in particular, which received fewer aggressions than females from the second year on.

Interestingly, our findings also confirm that, in addition to ravens' sex and age class, aspects of their fission–fusion dynamics can explain how strongly they engaged in conflicts. Specifically, we looked at the presence dynamics. In line with our hypothesis, we found ‘arriving’ and ‘local’ males to initiate higher rates of aggressions compared with ‘leaving’ males, which showed similar rates to females irrespective of their presence dynamic. We also found ‘arriving’ birds to receive high levels of aggressions, but only when juveniles. This latter finding also suggests that young ravens face the challenge of (re)integrating into local foraging groups, whereas older birds do not seem to have this problem any more.

Finally, we found the vagrant–resident index to positively correlate with the frequencies of initiated and received aggressions. This was expected since in this particular analysis fitting behavioural frequencies (objective 2), the index was basically a measure of the proportion of time in study (i.e. how often the bird was observed). But we also found that the vagrant–resident index had no effect when tested as a predictor of rank (objective 1). This indicates that the dominance status in this foraging community is independent from the frequency of visits to our specific study site. However, it does not necessarily mean that dominance status is independent from how often individuals meet with others. Indeed, ravens likely rely on multiple sites to forage, e.g. other anthropogenic food sources. It is thus likely that birds that encounter each other in our foraging site also meet in other locations [38]. Future studies will aim to investigate multiple neighbouring foraging sites to detect communities of individuals that meet more often than others, and analyse whether dominance rank is bound to a specific geographical location or a community of individuals.

### (c) Implications for cognition

Our surprising findings on dominance hierarchies suggest that wild ravens can cope with and keep track of a relatively large number of conspecifics on an individual basis, when competing for food resources. At our study site, the feedings of zoo animals serve as a strong attractor (more than 90% of all ravens present per day are seen at those feedings), but foraging bouts at enclosures are short (boars: 15–25 min, bears/wolves: 5–15 min) owing to inter- and intra-specific competition. Per foraging bout, an individual raven is confronted with 20–80 conspecifics. The identity of those may change over weeks as about 50% of ravens visit the feedings only from time to time (seen in fewer than 20% of observations), while about 40% are seen regularly (at 20–60% of observation sessions) and about 10% frequently (at more than 60% of observation sessions). Hence, even when daily foraging groups are small, the number of individuals encountered within a period of 1.5 years is relatively large. The inferred hierarchies in our population included around 50 marked birds per period, which were seen at least 10% of the time at the feedings. The fact that on average only half of the birds in the local foraging groups are marked suggests that ravens foraging at our study site might be able to deal with up to 100 conspecifics. Such estimates compare well with the extensive memory skills for conspecifics found in elephants [83], sheep [84] and dolphins [85], and are in line with the hypothesis that high degrees of fission–fusion dynamics may lead to improved memory skills [31].

Surprisingly, the daily affiliative ratio (amount of affiliations initiated and received) did not seem to predict individual rank or how much they received aggressions. However, besides individuals’ attributes (sex and age) and presence dynamics, we found that, to a lesser extent, birds' affiliative status also explains how much they initiate aggressions. Birds with a higher daily affiliation ratio (thus with more numerous or stronger potential allies) tended to initiate more aggressions than birds with a lower ratio. This corroborates that after sex and age, bonding status is another predictor for the outcome of dyadic conflicts in ravens [37], although it might not have a strong impact on rank. It also fits previous findings that older bonded ravens tend to intervene in affiliations between younger ravens (potentially in the process of forming a new strong bond), and doing so might prevent them from becoming future competitors [29]. Additionally, whenever a raven is engaged in a social bond (mated partner and/or affiliate) its chances of winning a fight, increase dramatically, while increasing bond strength further increases the likelihood of winning a fight with or without the presence of the partner [37]. Note, that we used the daily ratio of affiliations to approximate bonding status (i.e. type and number of relationships). If it may be reliably assessed for wild populations (with all identities known, and every single interaction tracked down), we could expect to find a more significant impact on rank and agonistic patterns, in particular for territorial breeders (in the minority in foraging groups, thus for which the effect might have been diluted using the affiliation ratio).

Our results pose the question of categorization of ranks, and whether ravens might categorize dominance ranks based on sex, age class and eventually bonding status [37]. Individuals would then only need to remember the actual ranks and rank differences of individuals within their own category (e.g. male/adult and eventually bonded). Such a cognitive ‘strategy’ would, however, predict a step-wise pattern in the dominance hierarchy, where the steps demarcate different sex, age classes and bonding categories, and linear rank orders within each step. Instead, we find in both wild populations one overall steep and transitive hierarchy, encompassing all sexes and age classes. This suggests that using individual attributes and behavioural heuristics alone does not suffice. Furthermore, experimental results from simulated (playback) encounters indicate that captive ravens are capable of mentally representing others' rank relationships [28]. Captive ravens even respond to simulated rank changes from adjacent aviaries, indicating that they can infer third-party relationships by observations only, i.e. without being able to compare ranks with their own rank position [28]. As the bonding status of ravens, and especially of subadult ravens without a territory, can be volatile [37,86], any heuristics would further need regular updating. To that effect, ravens may use transitive inference, as has been experimentally demonstrated in closely related pinyon jays [14]. Future studies on third-party interactions in a dynamic setting should aim to further our understanding of the strategical use of third-party knowledge in this species and its consequence on the dominance structure(s).

## 5. Conclusion

To conclude, our results indicate that in the wild, ravens can form and maintain dominance relationships with a large number of conspecifics despite the open and dynamic nature of their foraging groups. These relationships are the backbone of a steep and transitive hierarchy, which encompasses all sexes and age classes. Although the fission–fusion dynamic in this species might alleviate the costs of competition, via the adjustment of parties' size and composition, it does not seem to prevent the establishment of a complex social structure, apparently resilient to constant demographic changes. On the contrary, ravens seem to be able to fine-tune their behaviour to their presence dynamics. In line with ravens’ renowned cognitive skills, this suggests that the high unpredictability and variability of their social environment do not hinder them from using their skills but, instead, open up opportunities for advanced socio-cognitive mechanisms.

## Data accessibility

The processed datasets and R code supporting this article have been uploaded as part of the electronic supplementary material.
